# Glucosinolate-derived isothiocyanates impact mitochondrial function in fungal cells and elicit an oxidative stress response necessary for growth recovery

**DOI:** 10.3389/fpls.2015.00414

**Published:** 2015-06-03

**Authors:** Benoit Calmes, Guillaume N’Guyen, Jérome Dumur, Carlos A. Brisach, Claire Campion, Béatrice Iacomi, Sandrine Pigné, Eva Dias, David Macherel, Thomas Guillemette, Philippe Simoneau

**Affiliations:** ^1^Université d’Angers, INRA, Agrocampus Ouest, UMR 1345 IRHS, SFR 4207 QUASAVAngers, France; ^2^Universitatea de Ştiinţe Agronomice şi Medicinǎ Veterinarǎ BucureştiBucharest, Romania

**Keywords:** *Alternaria brassicicola*, isothiocyanates, oxidative stress, mitochondria, ROS

## Abstract

Glucosinolates are brassicaceous secondary metabolites that have long been considered as chemical shields against pathogen invasion. Isothiocyanates (ITCs), are glucosinolate-breakdown products that have negative effects on the growth of various fungal species. We explored the mechanism by which ITCs could cause fungal cell death using *Alternaria brassicicola*, a specialist *Brassica* pathogens, as model organism. Exposure of the fungus to ICTs led to a decreased oxygen consumption rate, intracellular accumulation of reactive oxygen species (ROS) and mitochondrial-membrane depolarization. We also found that two major regulators of the response to oxidative stress, i.e., the MAP kinase AbHog1 and the transcription factor AbAP1, were activated in the presence of ICTs. Once activated by ICT-derived ROS, AbAP1 was found to promote the expression of different oxidative-response genes. This response might play a significant role in the protection of the fungus against ICTs as mutants deficient in AbHog1 or AbAP1 were found to be hypersensitive to these metabolites. Moreover, the loss of these genes was accompanied by a significant decrease in aggressiveness on *Brassica*. We suggest that the robust protection response against ICT-derived oxidative stress might be a key adaptation mechanism for successful infection of host plants by Brassicaceae-specialist necrotrophs like *A. brassicicola*.

## Introduction

Plants- as part of their overall defense arsenal- counter pathogen attack by producing *de novo* antimicrobial phytoalexins (Hammerschmidt, 1999; Ahuja et al., 2012). Besides these newly synthesized metabolites, many plant species also constitutively accumulate compounds referred to as phytoanticipins (Osbourn, 1996) that, due to their high concentration in tissues and potential antimicrobial activities, may also contribute to protecting the plant from pathogen infection. For instance, it has been reported that falcarinol-type polyacetylenes accumulate in carrot leaves at concentrations estimated as being 5- to 15-fold higher than IC_50_ values reported for the fungal pathogen *Alternaria dauci* (Lecomte et al., 2012). In the same vein, garlic can yield ∼2 mg g^-1^ of the thiosulfinate allicin (Slusarenko et al., 2008) while sevenfold lower quantities are sufficient to inhibit the growth of numerous fungal pathogens (Curtis et al., 2004). Phytoanticipins are a very heterogeneous group of molecules with high structural diversity and the basis of their biocidal activity on fungal cells may not be unique. Indeed, the toxicity of the major oat root saponin avenacin has been associated with its ability to form complexes with fungal membrane sterols, leading to pore formation and loss of membrane integrity (Morrissey and Osbourn, 1999). Similarly, falcarindiol might induce permeabilization of the fungal plasma membrane (Lecomte et al., 2012). By contrast, allicin is readily taken up by fungal cells and, due to its oxidizing properties, might activate apoptosis (Gruhlke et al., 2010).

Members of the Brassicaceae plant family constitutively accumulate high levels (up to 1% of dry weight) of sulfur-containing glucosides called glucosinolates (GLS; Fahey et al., 2001). Upon tissue damage (e.g., during pathogen invasion), GLS are enzymatically converted into various breakdown products. Isothiocyanates (ITCs) are one of these myrosinase-catalyzed hydrolytic products (Lambrix et al., 2001) which have been shown to inhibit the growth of various pathogens *in vitro* (Tierens et al., 2001; Sellam et al., 2007a). *In planta*, the protective role of GLS against pathogen invasion has yet to be clarified but their catabolism could yield products able to confine fungal infection via their cytotoxicity or through activation of innate immune responses (Bednarek et al., 2009; Clay et al., 2009; Stotz et al., 2011). The cell toxicity of ITCs has mainly been studied on mammal cells due to their antitumor activity. They indeed have the capacity to inhibit the growth of several types of cancer cells by causing apoptotic and autophagic cell death (Cuddihy et al., 2008; Mi et al., 2008; Boreddy et al., 2011). The mechanism by which ITCs causes cell death is not yet fully understood and they may mediate their effects either via direct protein modification or indirectly by disruption of redox homeostasis and increased thiol oxidation (Brown and Hampton, 2011). In line with this, it has been shown that following exposure to ITC, fungal cells displayed a response similar to that elicited during oxidative stress with over-expression of several genes potentially involved in cell protection against oxidative damage (Sellam et al., 2007b).

The aim of this study was to further explore the mechanism by which ITCs exert their toxicity on fungal cells and to study the adaptive response of a Brassicaceae- specific fungus. We used as a model *Alternaria brassicicola*, the causal agent of the black spot disease of Brassicaceae, to dissect the effects of these glucosinolate-breakdown products on mitochondrial function, intracellular accumulation of reactive oxygen species (ROS) and oxidative stress signaling.

## Materials and Methods

### Fungal Strains and Growth Conditions

The *A. brassicicola* wild-type (WT) strain *Abra43* used in this study has previously been described (Dongo et al., 2009; Joubert et al., 2011). For routine culture*, A. brassicicola* was grown and maintained on potato dextrose agar (PDA). The method based on micro-scale liquid cultivation (from conidial suspensions) and automated nephelometric recording of growth, followed by extraction of relevant variables (lag time and growth rate), was described by Joubert et al. (2010). To study the susceptibility of fungal strains to ITC, allyl-ITC (AlITC), benzyl-ITC (BzITC), or phenetyl-ITC (PhITC), all purchased from Aldrich Chemical Co. (Milwaukee, WI, USA), were diluted from stock solutions prepared in methanol at the final desired concentrations. Solvent concentrations in controls and assays did not exceed 1% (v/v).

### RNA Isolation and Expression Analysis by Real-Time Quantitative PCR

Total RNA was prepared according to the TRIzol reagent protocol (Invitrogen). Additional cleanup and DNase treatment were performed using the Nucleospin RNA II kit (Macherey-Nagel) according to the manufacturer’s protocol. First-strand complementary DNA was synthesized from 5 μg of total RNA and used for real-time PCR. Amplification experiments were conducted as previously described (Sellam et al., 2007b) with specific primer combinations (Supplementary Table S1). The relative quantification analysis was performed using the comparative ΔΔCt method as described by Winer et al. (1999). To evaluate the gene expression level, the results were normalized using Ct values obtained from tubulin cDNA amplifications run on the same plate.

### Generation of Targeted Gene Replacement Constructs and Fungal Transformation

The construction of the Δ*abhog1* strain was previously described by Joubert et al. (2011). To construct the *Δabap1* mutant strains, a gene replacement cassette was generated using the double-joint PCR procedure (Yu et al., 2004). The selectable marker inserted in the PCR constructs corresponded to the *Hph* gene cassette (1436 bp) from pCB1636 (Sweigard et al., 1995) which confers resistance to hygromycin B. The sets of primers used to amplify the 5′ and 3′ flanking regions of the targeted gene are presented in Supplementary Table s1. The double-joint final PCR products were used to transform *A. brassicicola Abra43* protoplasts as described by Cho et al. (2006). Potential transformants were prescreened by PCR with relevant primer combinations (Supplementary Table S1) to confirm integration of the replacement cassette at the targeted locus. Two putative gene replacement mutants were further purified by three rounds of single-spore isolation and then confirmed by PCR and Southern blot analysis. Genomic DNA extraction and Southern blot analysis were conducted as previously described by Joubert et al. (2011).

### Generation of Fusion Protein Constructs

The AbHog1 and AbAp1 C-terminal GFP fusion constructs were generated by fusion PCR as described in Pochon et al. (2013). Using *A. brassicicola* genomic DNA as template, the respective ORFs and 3′ flanking regions were amplified with relevant primer combinations (Supplementary Table S1). In parallel, a fragment containing the sGFP and Hyg B cassettes were amplified from the plasmid pCT74 (Lorang et al., 2001) and pCB1636, respectively. The resulting PCR fragments were mixed and subjected to second fusion PCR. A linker containing three glycine residues was introduced at the 3′ end of the respective ORFs to replace the stop codons. The final PCR products were transformed either in the *A. brassicicola* WT strain *Abra43* or in a derivative strain constitutively expressing mCherry-NLS from plasmid pBV579 (Khang et al., 2010) under control of the *ToxA* promoter from pCT74. Transformants with the expected genetic integration events were identified by PCR.

### Infection Assays

For plant infection assays on *Brassica oleracea* plants (var. Bartolo), 5 μL drops of *A. brassicicola* conidia suspension (10^5^ conidia/mL in water) with or without diphenyleneiodonium (DPI; 0.4 μM) dissolved in DMSO were inoculated on leaves from 5 weeks-old plants. Inocula were symmetrically deposited on the left and right sides of the central vein. The plants were then maintained under saturating humidity (100% relative humidity). Symptoms were monitored at 7 days post-inoculation (dpi). Ten leaves were inoculated per condition and the experiment was repeated twice.

### Western Blot Analysis

The phosphorylation status of Hog1-related MAPK in *A. brassicicola* was studied by western blot using antibodies directed against dually phosphorylated forms of p38 MAPK (Cell Signaling Technology, Beverly, MA, USA). Total Hog1 proteins were detected using anti-Hog1 antibodies (Santa Cruz Biotechnology). Samples for study of the Hog1-related protein phosphorylation status were prepared from mycelia obtained by growing conidial suspensions at 25°C for 24 h in PDB (2 × 10^5^ conidia/mL) and then exposed to 2.5 mM AlITC. Mycelia were collected by filtration on filter paper, ground with a mortar and pestle to a fine powder under liquid nitrogen and homogenized in ice-chilled buffer containing protease and phosphatase inhibitors [50 mM Na phosphate, pH 7.4, 1 mM EDTA, 5% (v/v) glycerol, 1 mM PMSF, 50 mM NaF, 5 mM Na pyrophosphate, 0.1 mM Na vanadate, 10 mM *b*-glycerophosphate]. Extracts were centrifuged at 10 000 *g* for 10 min, and the resulting supernatants were stored at -80°C until use. The protein concentration in the extracts was calculated using a BCA protein assay reagent (Pierce, Rockford, IL, USA). Equal quantities (5 μg) of protein samples were loaded on 10% polyacrylamide gels and blotted onto nitrocellulose membranes (Schleicher and Schuell, Dassel, Germany). For each treatment, protein samples were prepared from at least three independent cultures and each sample was used to prepare at least three series of duplicated blots. Antibody binding was visualized using an ECL Plus Western blotting detection reagent (Amersham Biosciences, Buckinghamshire, UK) after binding of a horseradish peroxidase-conjugated secondary antibody.

### Intracellular Detection of Oxidative Products

Intracellular ROS were detected on 16 h-old germinating conidia after exposure to 2.5 mM Al-ITC or 1% (v/v) methanol for 1 h. After treatment, the incubation mixtures were mixed with 2′,7′-dichlorodihydrofluorescein diacetate (H_2_DCF-DA, Molecular Probes) or dihydroethidium (DHE, Molecular Probes) solutions (1 μM final concentration) and observations were performed under a fluorescent microscope (Leica DM4500) with the following filter combinations: 546 and 605 nm excitation and emission wavelengths respectively for DHE or 480 and 527 nm excitation and emission wavelengths respectively for H_2_DCF-DA.

### Measurement of Mitochondrial Transmembrane Potential

To measure the change in mitochondrial transmembrane potential (ΔΨm), 16 h-old germinating conidia were treated for 10 min with 2.5 mM Al-ITC or 1% (v/v) methanol and then the cationic lipophilic dye 5,5′,6′-tetrachloro 1,1′,3,3′tetraethylbenzimidazolylcarbocyanine iodide (JC-1; Invitrogen) was added (2 μg/mL final concentration), and the mixture was further incubated for 10 min in the dark. Fungal mats were then collected by filtration, washed thoroughly with PDB and observed under a fluorescent microscope with the following filter combinations: 546 and 605 nm excitation and emission wavelengths for the visualization of JC-1 aggregates in mitochondrial matrix, or 480 and 527 nm excitation and emission wavelengths for visualization of monomers in the cytoplasm of cells with depolarized mitochondria. To calculate the green/red fluorescence ratio of JC-1, images were all acquired with the same settings and exported in the software ImageJ^1^ to quantify pixel numbers corresponding to green and red fluorescence.

### Oxygen Consumption Rate Measurement

The respiratory activity was measured using the MitoXpress (Luxcel Biosciences, Cork, Ireland) fluorescent probe. Conidia (10^4^/mL) of WT *Abra43* were germinated for 14 h at 25°C in PDB medium, in the wells of a 96-well plate, and growth was monitored with nephelometry. The medium was then removed by aspiration and replaced with 150 μL of diluted (10% v/v) PDB medium containing 100 nM MitoXPress, and overlaid with 100 μL mineral oil. Inhibitors (Al-ITC or KCN) were added at this stage. Oxygen depletion in the medium was estimated from the increase in the fluorescence lifetime (LT) of the probe using a Fluostar Omega plate spectrofluorometer equipped with a time-resolved fluorescence head (BMG LABTECH GmbH, Ortenberg, Germany). At each time point, fluorescence relative units (340 nm excitation, 605–705 nm emission) were recorded (50 flashes) for 30 μs after 30 and 70 μs, and the probe LT was calculated from the fluorescence intensity ratio at 30 and 70 μs delays as follows: LT = (70-30)/Ln (W1/W2) where 70 is the W2 delay time (70 μs) and 30 is the W1 delay time (30 μs). W1 is the RFU signal measured after the W1 delay, W2 is the RFU signal measured after the W2 delay. MitoXpress LT data were analyzed using the MARS data analysis software version 2.30 (BMG LABTECH GmbH, Ortenberg, Germany). To assess the impact of Al-ITC or KCN on hyphae respiration, the slopes of LT were measured 60–90 min after the addition of the compounds, and compared to those of the control.

## Results

### Susceptibility of *A. brassicicola* to ITC

**FIGURE 1:**
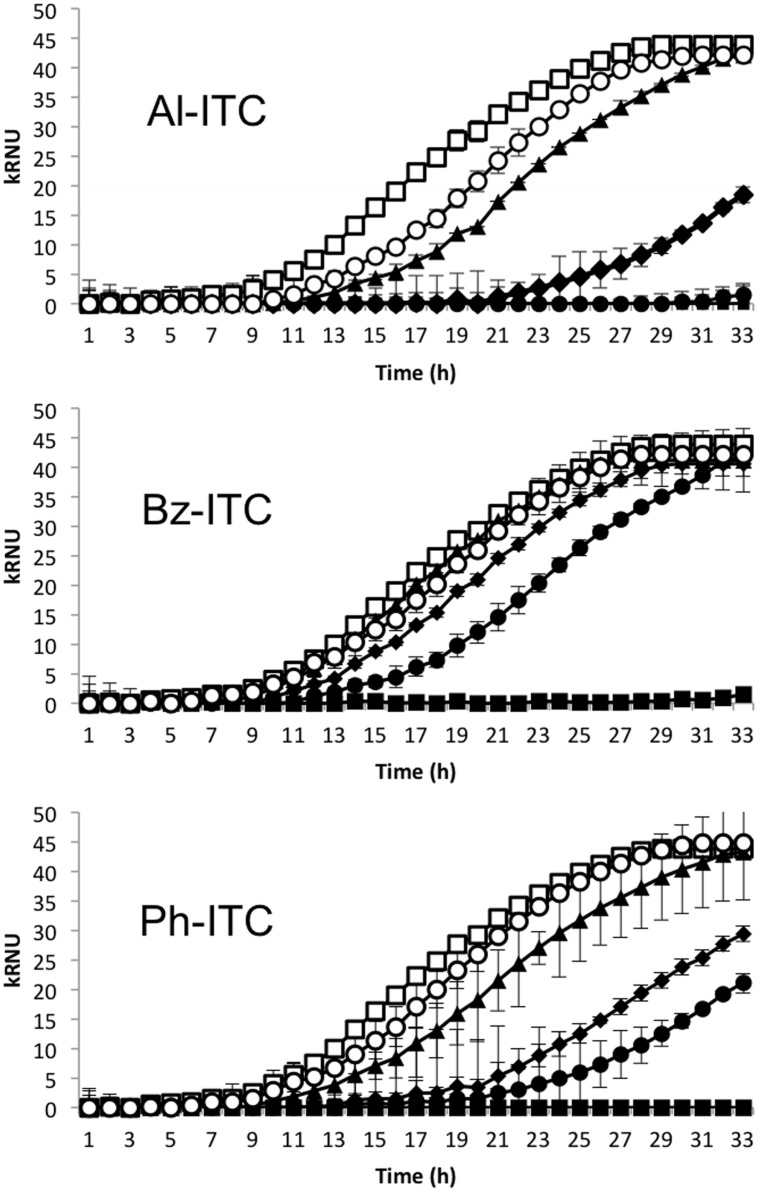
**Susceptibility of *Alternaria brassicicola* to allyl-, phenetyl-, and benzyl-isothiocyanates.** Nephelometric monitoring of the growth of wild-type (WT) strain *Abra 43* was automatically recorded for 33 h at 24°C. The *Y*-axis data correspond to the relative nephelometric units (RNUs). Conidia were used to inoculate microplate wells containing standard PDB medium that was supplemented with 1.25 mM (open circles), 2.5 mM (black triangles), 5 mM (black diamonds), 10 mM (black circles), 20 mM (black squares) of each isothiocyanate, or methanol (positive control; open squares). Each condition was tested in triplicate and the experiments were repeated twice. The areas under the curves were calculated from the growth curves and used to calculate IC_50_.

Analyses of growth curves in liquid medium supplemented with various concentrations of Al-ITC, Bz-ITC, or Ph-ITC were used to assess the susceptibility of the WT *A. brassicicola* strain *Abra43* to different ICTs. Areas under the curves were used to estimate the growth inhibitory effect of each compound and calculate the IC_50_. As shown in **Figure 1**, maximal and minimal inhibitions were obtained with Al-ITC (IC_50_ 2.9 mM) and Bz-ITC (IC_50_ 6.0 mM), respectively. An intermediate effect was observed with Ph-ITC (IC_50_ 4.2 mM). Due to the higher toxic effect of Al-ITC on *A. brassicicola*, most of the experiments were then performed using this compound. Careful examination of the growth curves revealed that the sensitivity of *A. brassicicola* to the different ITCs was clearly explained by a delayed entry into the log phase (i.e., increased lag times) while its maximum growth rate was only slightly affected.

### ITC Induces Intracellular ROS Accumulation

**FIGURE 2:**
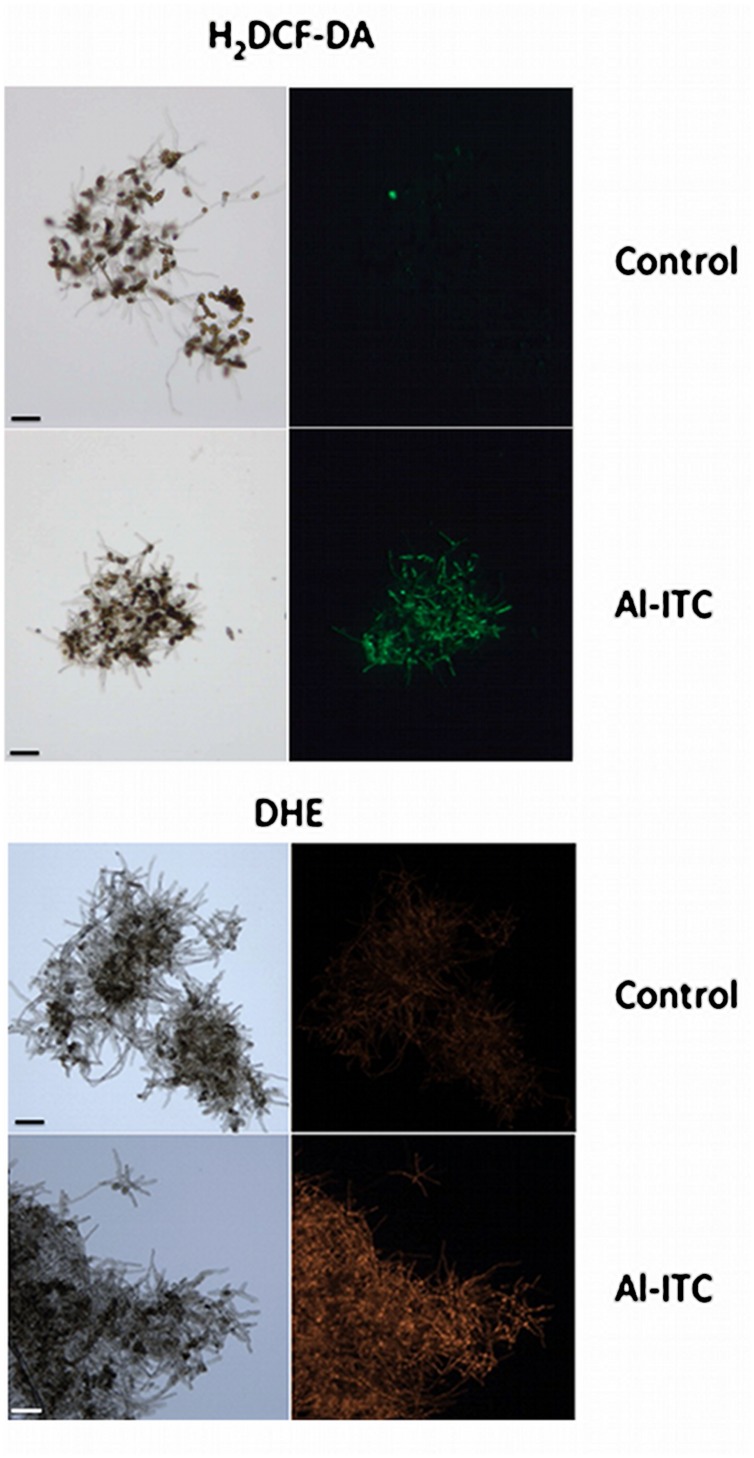
**Visualization of oxidative stress symptoms in *A. brassicicola* treated with allyl-isothiocyanates.** ROS within hyphae of germinated conidia treated for 1 h with methanol 1% v/v (control) or 2.5 mM Al-ITC were detected using the fluorescent dyes H_2_DCFDA and DHE. For each panel, the right part corresponds to fluorescence microscopy and the left part to bright-field microscopy. Scale bars = 50 μm.

Reactive oxygen species generation of fungal cells exposed to 2.5 mM Al-ITC was monitored by using H_2_DCFDA, a cell-permeable general ROS indicator that penetrates live cells but does not fluoresce unless oxidized by ROS. Intense green fluorescence was distributed along the hyphae in ITC-treated sample, indicating that ROS was generated in the cells. No ROS- specific signals were detected in control hyphae (**Figure 2**). This result was confirmed after visualization of ROS production by incubating cells with DHE, a non-fluorescent compound which, upon reacting with superoxide, is converted to a fluorescent derivative. These observations demonstrated that ITC promoted intracellular ROS production. Similar observations were obtained after exposure to Bz- and Ph-ITC (not shown).

### Hog1 MAP Kinase Activated Upon Exposure to ITC

**FIGURE 3:**
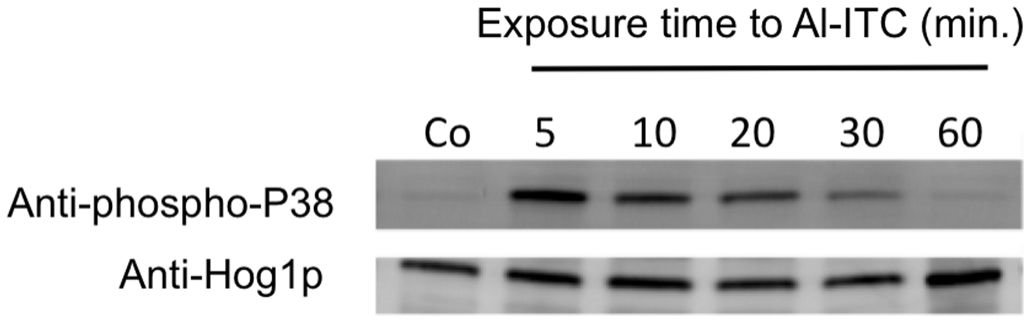
**Phosphorylation of the Hog1-like MAPK in *A. brassicicola* after exposure to allyl-isothiocyanate.** Germinated conidia from the WT strain (*Abra43*) were grown for 24 h in PDB and exposed to 2.5 mM Al-ITC for 5–60 min. Control (Co) cultures were supplemented with methanol alone. Total protein extracts, prepared from the harvested mycelia, were analyzed by SDS-PAGE and blotting with either anti-Hog1 C-terminus antibody or anti-dually phosphorylated p38 antibody.

**FIGURE 4:**
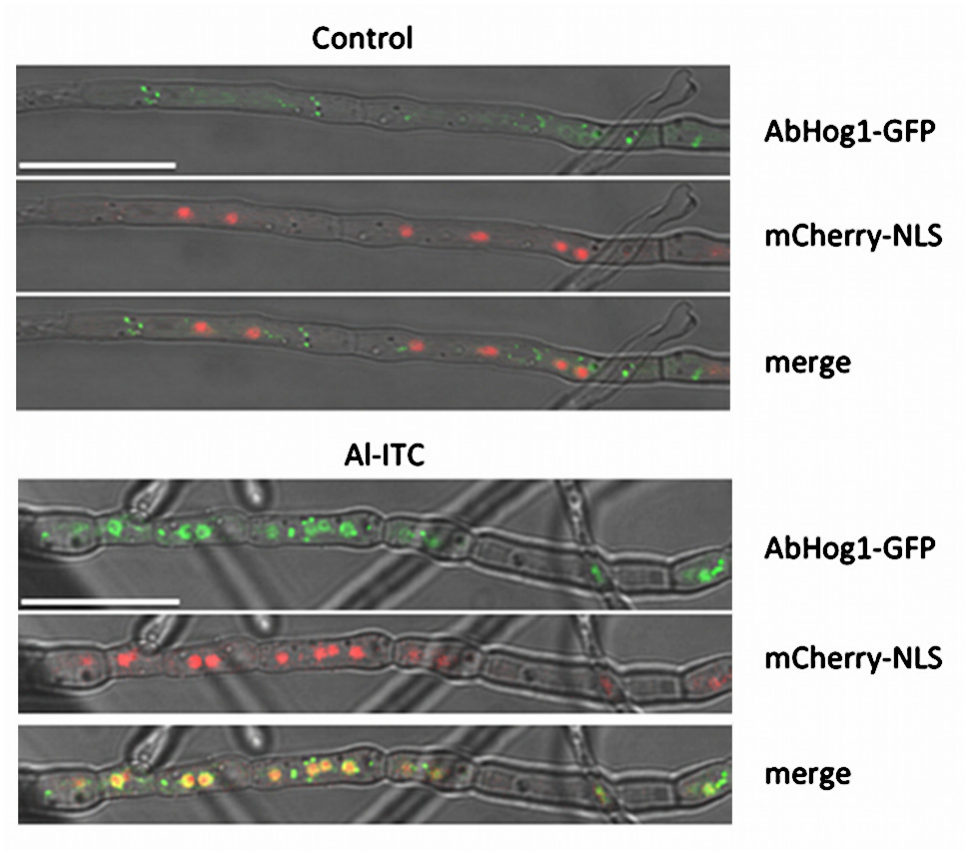
**Isothiocyanate-induced nuclear accumulation of the AbHog1-GFP fusion protein. Double-labeled strains expressing AbHog1-GFP and mCherry-NLS were exposed to either methanol (control) or 2.5 mM Allyl-ITC for 20 min**. Co-localization analyses were examined using confocal microscopy. Bars = 25 μm.

In some fungal species the MAP kinase Hog1 has been shown to be activated by ROS and actually mediate oxidative stress responses (Moye-Rowley, 2003; Aguirre et al., 2006; Lin and Chung, 2010). We thus explored the phosphorylation status of AbHog1 in *A. brassicicola* after exposure to Al-ITC using a western blot approach. *A. brassicicola* cells were grown in liquid medium, exposed for various times to Al-ITC and then harvested for total protein extraction. Immunoblot analysis using anti-phospho-p38 antibodies revealed that increased phosphorylation of the AbHog1 MAPK occurred as early as 5 min after exposure (**Figure 3**). Longer exposures resulted in a decreased signal and after 1 h over-phosphorylation of AbHog1 compared to the control was no longer observed. In response to various stresses, the activated Hog1 protein accumulated in the nucleus (Reiser et al., 1999). To test whether this phenomenon also occurs in *A. brassicicola* after exposure to ITC, a strain constitutively expressing the mCherry-NLS protein from the *ToxA* promoter and expressing a Hog1::eGFP fusion protein from the *AbHog1* promoter was constructed. Under control conditions this strain showed green fluorescence signals within the hyphae which did not co-localize with the red labeled fluorescence observed in nuclei (**Figure 4**). However, when cells were exposed for 20 min to 2.5 mM ITC dense green fluorescent spots were observed along the hyphae. Their distribution matched the labeled nuclei, thus demonstrating nuclear migration of the MAP kinase in ITC-treated cells.

### AbAP1 Transcription Factor Activated and Controlled Oxidative Response Gene Expression after ITC Exposure

**FIGURE 5:**
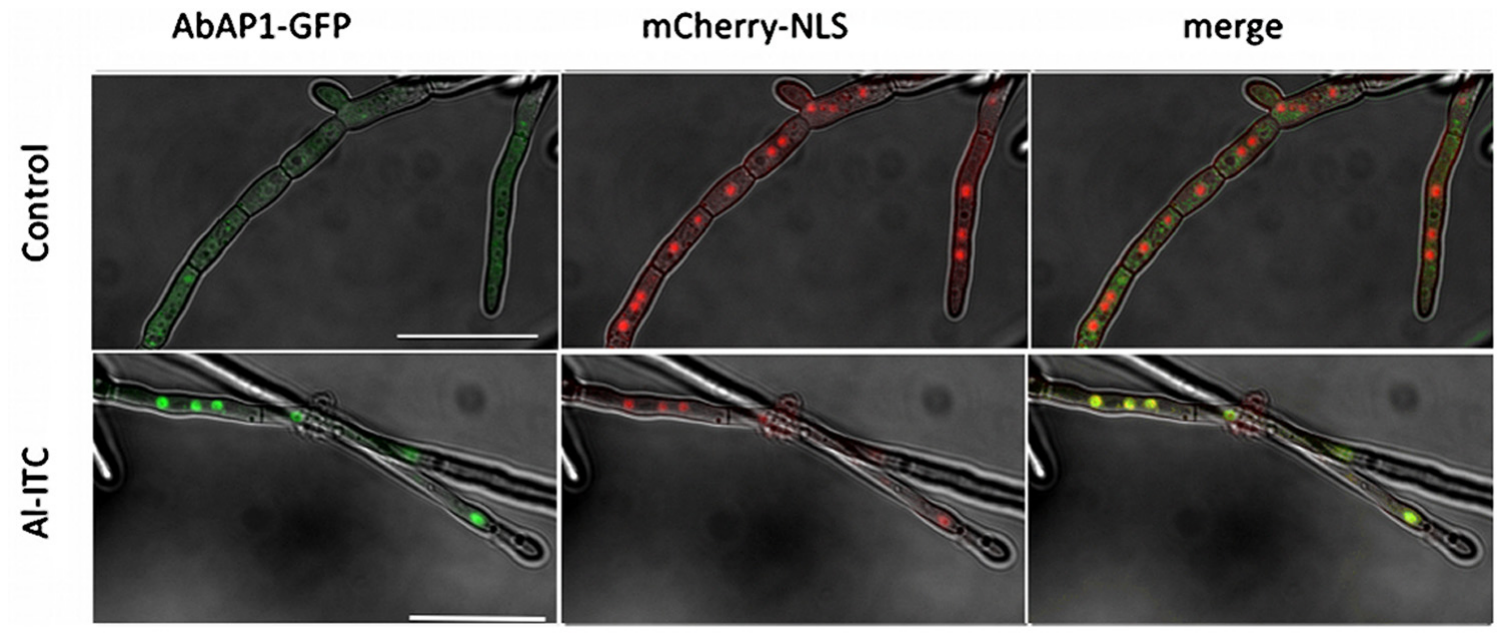
**Isothiocyanate-induced nuclear accumulation of the AbAP1-GFP fusion protein**. Double-labeled strains expressing AbAP1-GFP and mCherry-NLS were exposed to either methanol (control) or 2.5 mM Allyl-ITC for 20 min. Co-localization was examined using confocal microscopy. Bars = 25 μm.

*Saccharomyces cerevisiae* YAP1 protein and other AP1-like fungal orthologs are considered as major oxidative stress response transcription factors (TFs). An ortholog of this protein was identified as AB04817.1 via BLAST analyses in the *A. brassicicola* automatically annotated genome database^2^ and named AbAP1. Analysis of AbAP1 showed the expected conserved bZIP DNA-binding, nuclear localization, N-terminal and carboxyl terminal cysteine-rich domains (Supplementary **Figure S1**). AP1-like proteins behave like redox sensors that localize inside the nucleus upon exposure to ROS and then regulate the expression of a large set of genes including oxidative response genes (Lee et al., 1999; Lev et al., 2005; Asano et al., 2007; Temme and Tudzynski, 2009; Znaidi et al., 2009; Takahashi et al., 2010; Guo et al., 2011; Tian et al., 2011; Montibus et al., 2013). To determine whether this is also true for AbAP1 in response to ITC, we generated a strain co-expressing a C-terminal AbAP1::eGFP fusion protein and the mCherry-NLS protein. As shown in **Figure 5**, in the absence of oxidative stress, green fluorescence was distributed throughout the hyphae, suggesting cytoplasmic TF accumulation. By contrast after 20 min exposure to ITC, green fluorescence was concentrated in discrete spots co-localizing with the mCherry fluorescence signals indicating nuclear localization of AbAP1::eGFP in response to ITC.

We then checked whether oxidative stress response genes, previously reported as overexpressed in *A. brassicicola* exposed to ITC (Sellam et al., 2007b), required AP1 for induction. Mutants deficient in this TF were generated by gene replacement and exposed to ITC. The expression levels of a set of 14 genes were then compared to those measured in the same conditions for the parental WT strain. As expected all the selected genes were strongly overexpressed in the *Abra43* WT strain after 20 min of exposure to Al-ITC (**Table 1**). By contrast, the majority of the selected genes (11/14) were no longer induced, or induced at a significantly lower level (*p* < 0.01) under the same conditions in the *Δabap1* mutant. The expression of only three genes encoding a putative thioredoxin, a cytochrome P450 monooxygenase and a glutathione *S*-transferase was found independent of AbAP1 for induction.

**Table 1:** Expression of oxidative stress response genes in the wild-type (WT) and Δ*abap1* strains after 20 min of exposure to Al-ITC.

Target sequence (GenBank acc. #)	Potential Function^a^	Fold induction in WT (±SD)^b^	Fold induction in Δ*abap1*/Fold induction in WT	*p*-Value (*t-*test)
A3H11 (DY542661)	TRX	26.2 ± 4.6	0.07	8E-04
A2G8 (DY542662)	TRX	107.6 ± 24	0.08	0.002
A3G5 (DY542663)	TRX	77.4 ± 23.4	0.25	0.013
A1B12 (DY542664)	TRX	116.2 ± 13	0.04	1E-04
A4D11 (DY542665)	TRR	12.8 ± 0.88	0.22	1E-04
A2H9 (DY542667)	QOX	22.44 ± 2.6	0.24	0.002
A3D2 (DY5426674)	CytP450	8.39 ± 1.9	0.47	0.02
A3D10 (DY542658)	GPX	10.36 ± 0.8	0.17	4.9E-05
A2F9 (DY542659)	GCS	25.95 ± 6.1	0.05	0.002
A2H5 (DY542653)	GST	39.23 ± 11.6	0.19	0.009
A2C10 (DY542656)	GST	21.57 ± 1.27	0.09	1.9E-05
A1F1 (DY542654)	GST	9.01 ± 2.2	0.77	0.165
A2C1 (DY542655)	GST	12.78 ± 2.5	0.04	0.001
A4D12 (DY542657)	GST	8.74 ± 1.27	0.12	4E-04

^a^TRX, thioredoxin; QOX, quinone oxidoreductase; CytP450, cytochrome P450 monooxygenase; GPX, glutathione peroxidase; GCS, ox*g*-glutamylcysteine synthetase; GST, glutathione ox*S*-transferase. ^b^First strand cDNAs were prepared from RNA samples extracted from germinated conidia either exposed to 2.5 mM allyl-isothiocyanate (Al-ITC) or methanol 1% (control) for 20 min and used as template for real-time PCR. For each gene, expression induction is represented as a ratio (fold induction) of its relative expression (studied gene transcript abundance/b-tubulin transcript abundance) in each inductive condition to its relative expression in the control. Each value is the mean of two independent experiments with the WT strain and two independent Δ*abap1* mutants, each with three replicates.

### *A. brassicicola* Strains with an Impaired Oxidative-Stress Response are Hypersensitive to ITC

**FIGURE 6:**
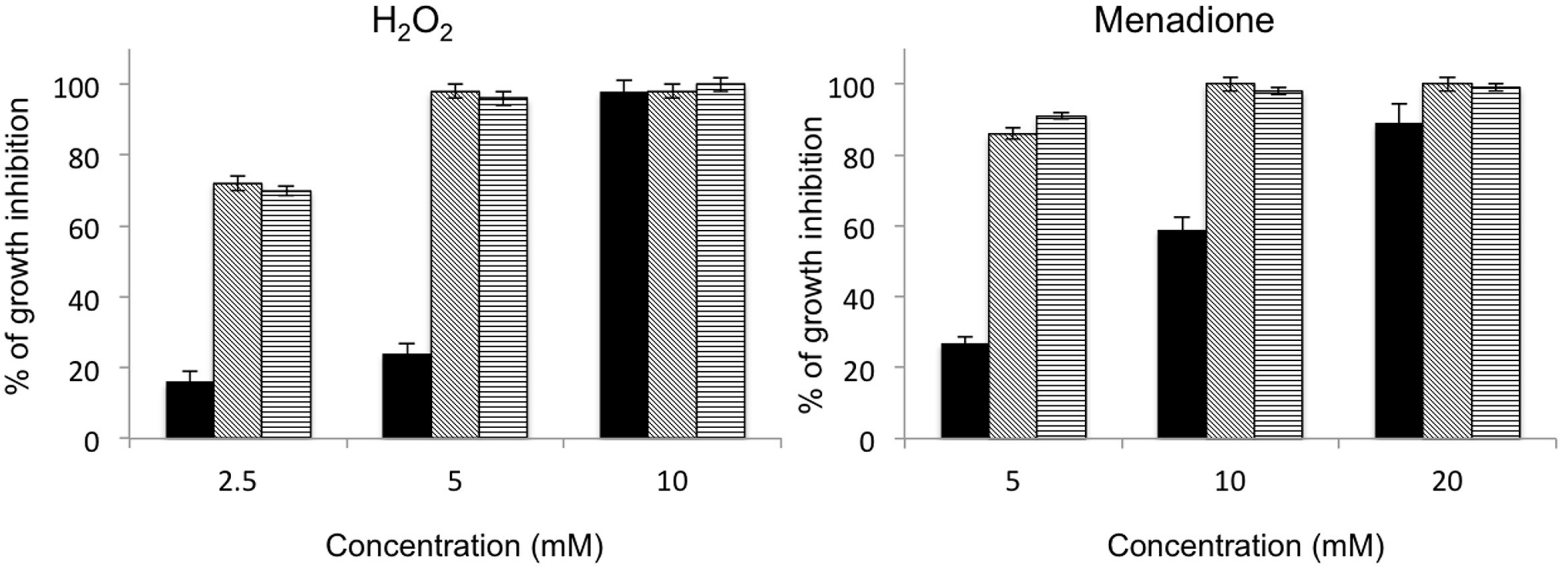
**Susceptibility of *A. brassicicola* wild-type (black bars), Δ*abhog1* (hatched bars) and Δ*abap1* (stripped bars) strains to oxidative stress**. Conidia of each genotype were used to inoculate microplate wells containing standard PDB medium supplemented with various concentrations of H_2_O_2_ or menadione. Nephelometric monitoring of growth was automatically recorded for 33 h at 24°C. Each condition was tested in triplicate and the experiments were repeated twice. The areas under the curves were used to calculate de percentages of inhibition for each treatment compared to control growth curves.

**FIGURE 7:**
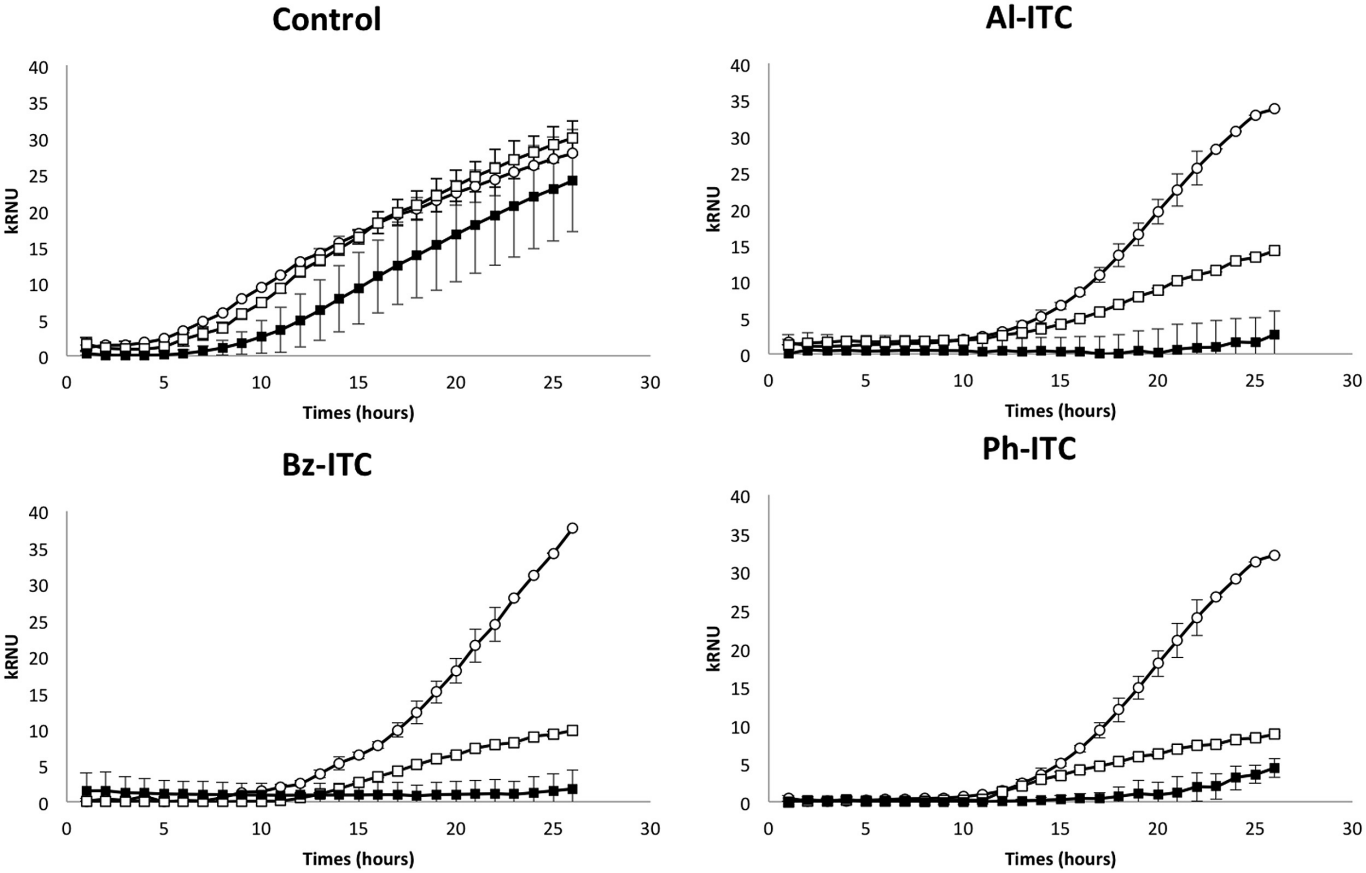
**Susceptibility of *A. brassicicola* WT (open circle), Δ*abhog1* (black square) and Δ*abap1* (open square) to allyl-, phenetyl-, and benzyl-isothiocyanates**. Nephelometric monitoring of growth was automatically recorded for 27 h at 24°C. The *Y*-axis data correspond to RNUs. Conidia were used to inoculate microplate wells containing standard PDB medium supplemented with either 5 mM Al-ITC or 5 mM Bz-ITC or 5 mM Ph-ITC or methanol (control). Each condition was tested in triplicate and the experiments were repeated twice.

Two Δ*abhog1* mutants (Joubert et al., 2011) and two Δ*abap1* mutants were tested for their susceptibility to different oxidizing compounds. Nephelometric monitoring of the initial growth stages was used to assess the effects of H_2_O_2_, menadione, and various ITCs (Al-, Bz-, and Ph-ITC) on the fungus. As independent strains behaved similarly for each genotype, the percentage of growth inhibition and growth curves shown in **Figures 6** and **7**, respectively, correspond to means of values obtained for individuals carrying the same mutation. Under control conditions (PDB medium), Δ*abhog1* mutant growth was slower than that of the WT and Δ*abap1* strains (**Figure 7**). All mutants were found to be more susceptible than the WT to the different treatments; Δ*abap1* and Δ*abhog1* mutants were highly susceptible to menadione and moderately susceptible to H_2_O_2_ (**Figure 6**). Exposure to all the tested ITCs dramatically affected Δ*abhog1* mutant growth and to a lesser extent that of Δ*abap1* mutants (**Figure 7**).

### ITC Disrupts the Mitochondrial Membrane Potential in Fungal Cells and Decreases Oxygen Consumption Rate

**FIGURE 8:**
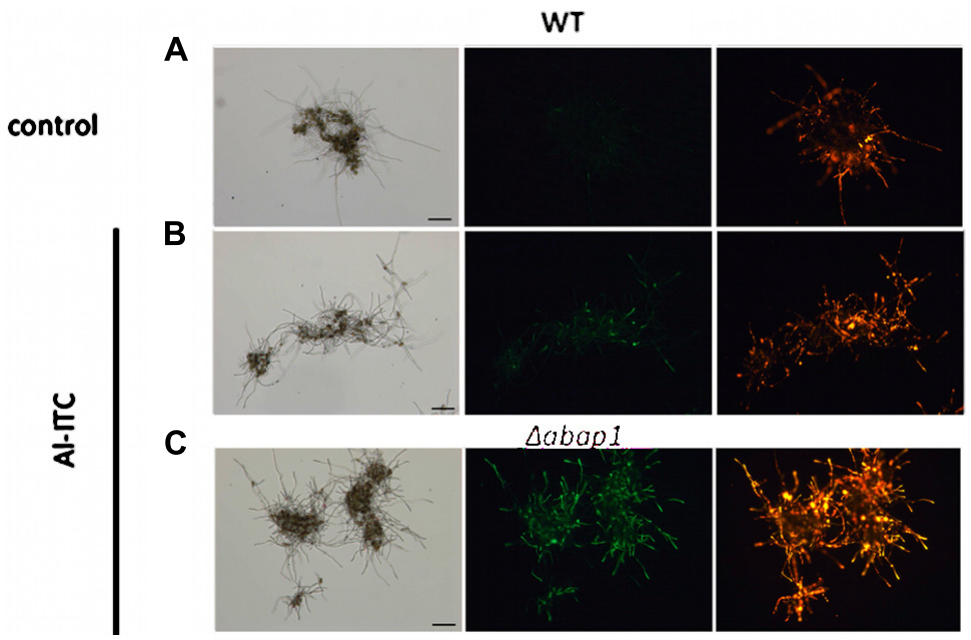
**Effect of allyl-isothiocyanate on the mitochondrial membrane potential of *A. brassicicola* cells**. The mitochondrial membrane potential was assessed within hyphae of germinated conidia from the WT strain *Abra43* **(A,B)** or the Δ*abap1* mutant **(C)** using the fluorescent potentiometric dye JC-1 after 10 min treatment with methanol 1% v/v (**A**, control) or 2.5 mM Al-ITC **(B,C)**. For panels **(A,B,C)** the left part corresponds to bright-field microscopy and the right parts to fluorescence microscopy. Red signals correspond to cells containing mitochondria with high membrane potential and green signals represent mitochondria with low membrane potential. Scale bars = 50 μm.

**FIGURE 9:**
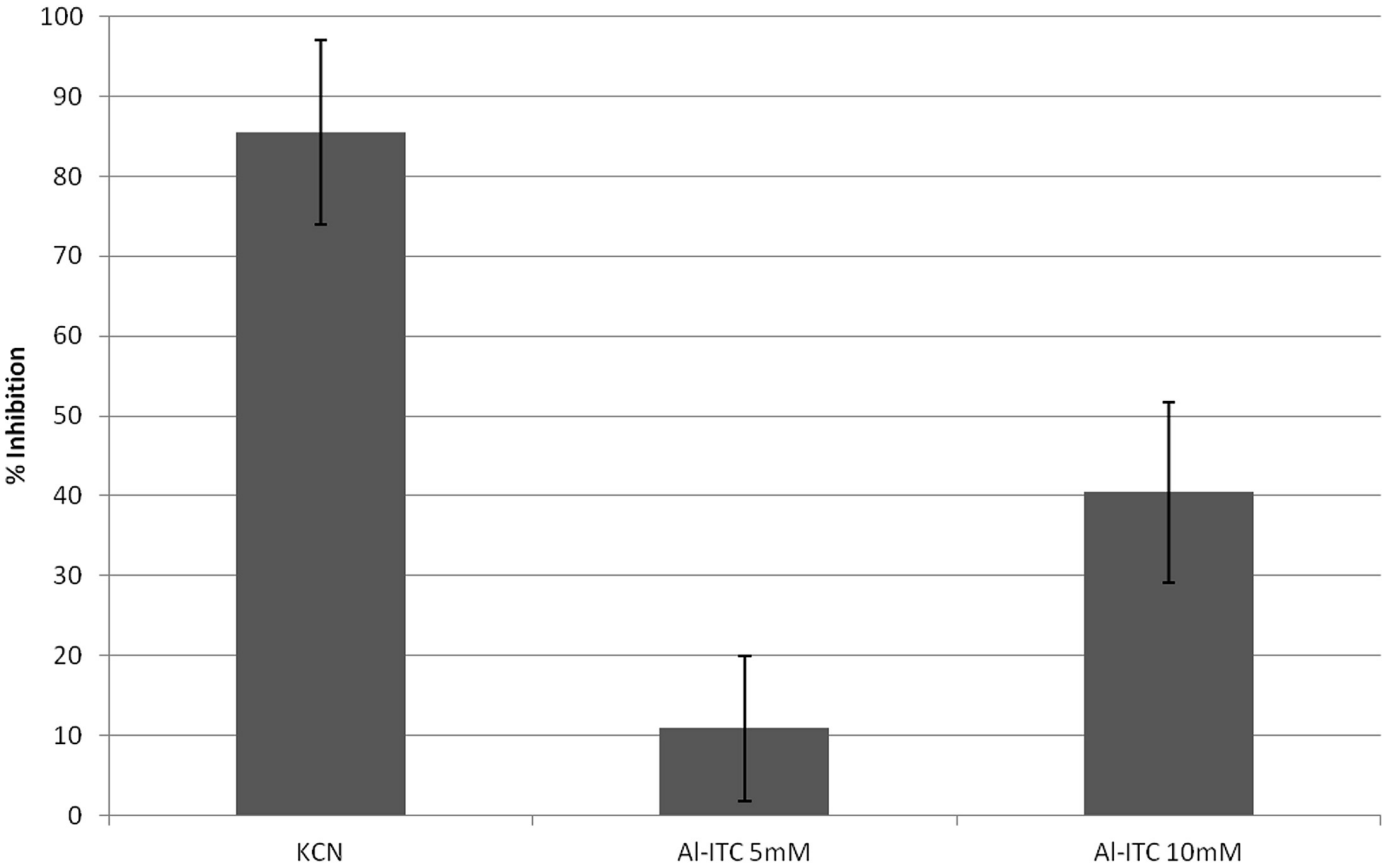
**Effect of Al-ITC on *A. brassicicola* hyphae respiration**. The graph shows the inhibitory effects (% of the control) of ITC or KCN on the respiration rate of 14 h germinated conidia, measured 60 min after the addition of the compounds. SD is indicated.

Various studies on human cells have assessed the effects of ICTs on mitochondria, and revealed that dissipation of the mitochondrial membrane potential (ΔΨ) occurs upon exposure. We tested whether a similar effect could be observed in fungal cells exposed to ITC using JC-1 dye. This cationic dye accumulates in the mitochondrial matrix as a function of the membrane potential. At high concentrations (reflecting a high ΔΨ) JC-1 forms aggregates displaying orange–red fluorescence. At lower concentrations, when ΔΨ decrease or collapse, JC-1 is present as monomers exhibiting a green fluorescence. The green/red fluorescence ratio of JC-1 is therefore an indicator of the mitochondrial membrane potential, and thus of mitochondrial function. After short-term exposure (10 min) to 2.5 mM Al-ITC, there was an increase in green fluorescence indicative of a decrease in mitochondrial ΔΨ (**Figure 8**). The mean green/red fluorescence ratio of the probe increased from 0.113 ± 0.037 in the control to 0.474 ± 0.021 after treatment. Under similar exposure conditions, monomeric JC-1 – associated green fluorescence in ITC-treated cells was more pronounced in the Δ*abap1* strain than in the WT with the mean green/red fluorescence ratio of the probe reaching 0.646 ± 0.095 (**Figure 8C**). These alterations in ΔΨ indicate that Al-ITC can rapidly induce modifications in mitochondrial functions and cellular energy metabolism. In order to further assess the impact of the compound on fungal respiration, oxygen consumption of hyphae was measured after 60 min exposure to Al-ITC using a water-soluble oxygen-sensitive fluorescent probe. In comparison to the untreated (solvent only) control, Al-ITC clearly inhibited respiration of the WT cells in a dose-dependent manner, reaching 40% inhibition at 10 mM (**Figure 9**). Cyanide, a strong inhibitor of cytochrome oxidase, induced 85% inhibition of respiration, suggesting low activity of the alternative oxidase in the WT strain (**Figure 9**).

### Mutants Defective in the Oxidative Stress Response have Decreased Aggressiveness on Glucosinolate Accumulating Host Plants

**FIGURE 10:**
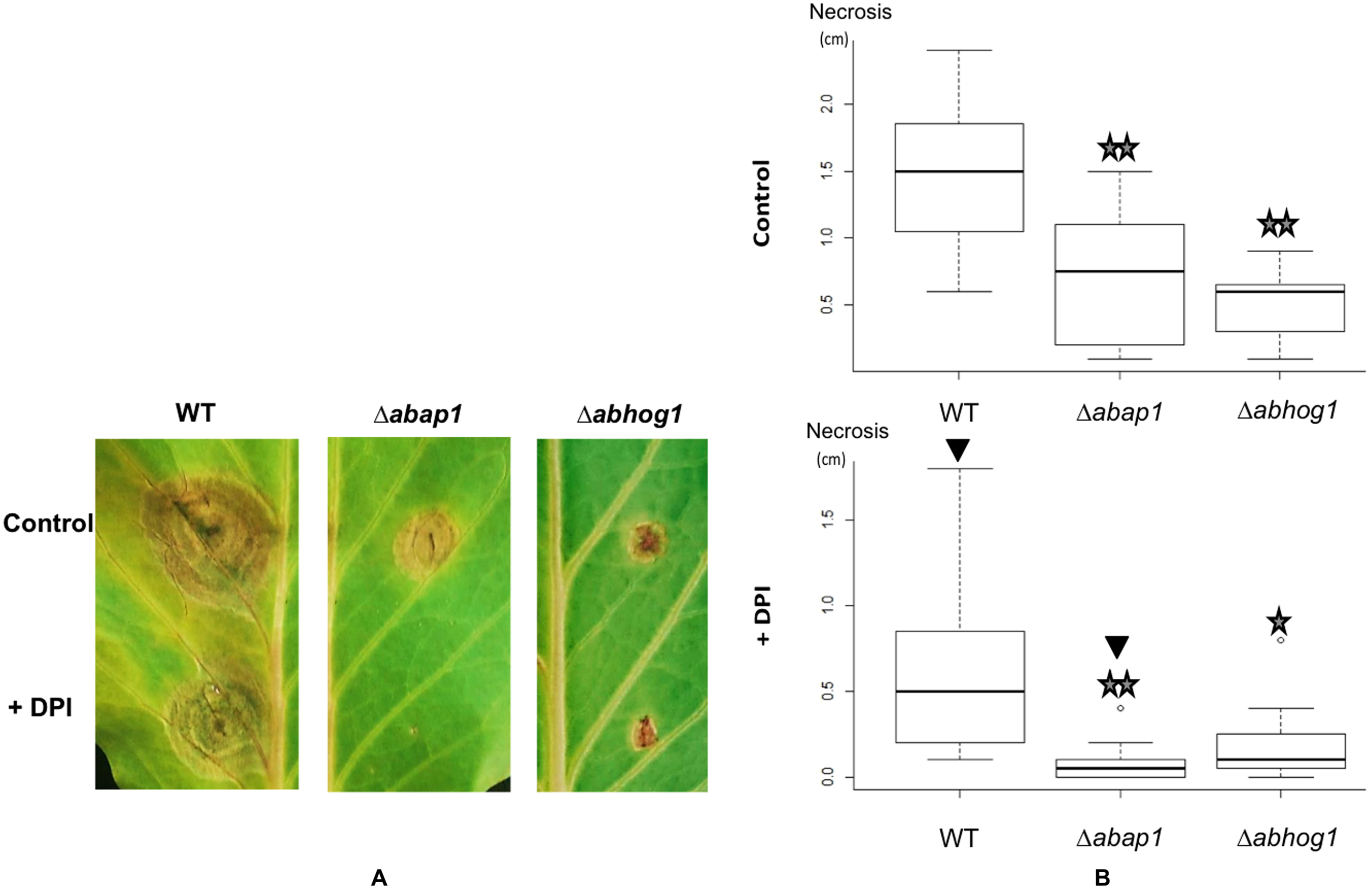
**Impact of *AbHog1* and *AbAP1* mutations on *A. brassicicola* aggressiveness.** WT and mutant strains were inoculated on cabbage leaves with or without (controls) the NADPH oxidase inhibitor DPI. Necrotic areas were observed and measured at 7 dpi. Photographs **(A)** represent typical symptoms for each genotype at 7 dpi. Necrotic areas were measured on 10 inoculated leaves per genotype and condition. On the graphs **(B)**, stars indicate a significant difference between mutant and WT strains using the Student test (*P* < 0.05 single star, *P* < 0.01 double star) and black triangles indicate a significant difference between the control and DPI treatment using the Student test (*P* < 0.01)

To test the effects of targeted *AbHog1* and *AbAP1* gene knockout on pathogenicity, *B. oleracea* leaves were inoculated with drops of conidia suspension (10^5^ conidia/mL) with or without the NADPH oxidase inhibitor DPI (0.4 μM). Necrotic areas were measured at 7 dpi. As shown in **Figure 10**, in the absence of DPI the virulence of Δ*abhog1* and Δ*abap1* mutants was significantly decreased compared to the WT strain. When the NADPH oxidase inhibitor was applied with the fungal inoculum significantly smaller necrotic areas were observed for all tested genotypes. At the selected concentration, DPI had no negative effect on conidia germination and hyphal growth. In such conditions, i.e., when oxidative stress generated *in planta* was mainly due to glucosinolate-derived ITCs, the Δ*abap1*Δ and the Δ*abhog1* mutants were almost completely non-virulent.

## Discussion

Glucosinolates are a class of S- and N- containing secondary metabolites that are found in only 15 botanical families of the Capparales order and are very abundant in the Brassicaceae family (Fahey et al., 2001). They are thought to play a variety of roles in plant defense responses. Indeed, it has been repeatedly reported that GLS-derived ICTs have a negative effect on the growth of various fungal species including specialist *Brassica* pathogens (Manici et al., 1997; Smolinska et al., 2003; Sellam et al., 2007a). It was previously reported that the inhibitory effect of ITCs depends on their type, and aliphatic ITCs usually have stronger *in vitro* inhibitory effects on fungi than aromatic ITCs (Smolinska et al., 2003). Moreover, it was shown that *Arabidopsis thaliana* mutants with low aliphatic GLS content had decreased ability to defend themselves against necrotrophic fungi (Stotz et al., 2011; Buxdorf et al., 2013). In line with these studies, we showed here that ITCs, at concentrations in the millimolar range, significantly slowed down the development of *A. brassicicola*, with allyl-ITC being much more efficient than phenetyl- and benzyl-ITC. These differences might reflect differential accumulation of ITCs in the fungal cell due to the side-chain structures or different mechanisms of action.

Many studies have explored the cellular targets of ITCs in mammalian cells that might explain their chemoprotective and anticarcinogenic properties (for recent reviews see Zhang, 2010; Navarro et al., 2011). By contrast, the mechanism by which they exert their toxicity on fungal cells has been poorly documented. Sellam et al. (2007b) conducted a transcriptomic analysis of the response of *A. brassicicola* to Al-ITC exposure. Among the overexpressed genes, more than one third could be considered as related to an adaptive response to cellular oxidative stress suggesting that the ITC mediates redox dysregulation in the fungal cell. Increased ITC-induced ROS accumulation was indeed demonstrated here using DHE and H_2_DCFDA probes. This observation corroborates the observed effects of ITC on many mammal cell lines such as human lung (Wu et al., 2010; Liu et al., 2012), breast (Xiao et al., 2008), and prostate (Xiao et al., 2010) cancer cells. In the latter study, it was demonstrated using the MitoSOX Red probe that ROS generation by ITC in cancer cells was mitochondria-derived. MitoSOX Red was previously reported to be a useful intracellular ROS indicator in different fungal species such as *Mycosphaerella graminicola* and *S. cerevisiae* (Batova et al., 2010; Scalliet et al., 2012). However, we did not succeed in localizing ROS accumulated in ITC-treated *A. brassicicola* cells using this fluorescent compound probably due to its weak penetration across the fungal cell-walls. Using JC-1 dye we clearly observed that ITC treatment of *A. brassicicola* disrupted the mitochondrial membrane potential. A similar observation was reported in breast cancer cells exposed to Bz-ITC (Xiao et al., 2006) and mitochondria were found to be the primary targets of ITCs in human bladder cells (Tang and Zhang, 2005). The question of whether the collapse of the mitochondrial membrane potential was caused by mitochondria-generated ROS has to be raised. In human breast and prostate cancer cells, it was indeed shown that ITC treatment reduced the oxygen consumption rate due to inhibition of complex III activity (Xiao et al., 2008, 2009). Using phosphorescence-quenching oxymetry, we showed here that exposure to a high concentration (10 mM) of Al-ITC also resulted in a significant decrease in the oxygen consumption rate by *A. brassicicola.* This suggested a possible effect on mitochondrial oxidative phosphorylation although inhibition of glycolysis or of the tricarboxylic acid cycle cannot be excluded.

Another mechanism by which ITC might disrupt cell redox homeostasis has been proposed and at least in part involves the glutathione cycle (Brown and Hampton, 2011). It has been suggested that these metabolites exacerbate oxidative stress by causing depletion of intracellular glutathione (Sahu et al., 2009; Wu et al., 2010; Tusskorn et al., 2013). GSH depletion is quite likely due to the inhibition of glutathione reductase (Hu et al., 2007) and increased conjugation of glutathione to ITC catalyzed by induced glutathione-*S*-transferases (Sahu et al., 2009). A similar mechanism has been proposed to explain the cell toxicity of another plant-derived thiol-selective reagent (Gruhlke et al., 2010). In line with this hypothesis, we showed here that the expression of five fungal GSTs was strongly induced after short-term exposure to ITC. However, it has yet to be determined whether these enzymes are all able to catalyze the conjugation of glutathione to ITC.

Irrespective of the mechanism by which ITC exerts its toxicity, exposure to even low concentrations of Al-ITC could thus potentially negatively impact the fungal cell metabolism, but interestingly, *A. brassissicola* seemed to manage the exposure to the compound (e.g., at 2.5 mM) since growth was delayed, but then proceed at an almost normal rate. This suggests that moderate concentrations of Al-ITC induce alterations in mitochondria (e.g., complex III inhibition) and/or depletion of intracellular glutathione, which results in increased ROS production. The latter elicits AbHog1- and AbAP1-mediated responses (and possibly others), which reinforce anti-oxidant mechanisms, allowing the energy metabolism to cope with the presence of Al-ITC.

In line with this hypothesis, the ITC-induced overexpression of the majority of the oxidative stress response genes analyzed in our study was found to be AP1-dependent. AP1-like TFs have been shown to have crucial roles in the regulation of the oxidative stress responses in yeast and filamentous fungi. These TFs induce, when activated, the expression of many antioxidants and related protein-encoding genes such as genes involved in the thioredoxin and glutathione systems (Temme and Tudzynski, 2009; Takahashi et al., 2010; Guo et al., 2011; Tian et al., 2011). For instance, in line with our findings, among the 12 menadione-induced genes identified as expressed in an AP1-dependent manner in *Neurospora crassa* by Takahashi et al. (2010) four were putative GSTs. Yeast AP1 and other fungal AP1-like proteins characterized so far are B-ZIP TFs whose subcellular localization is under redox control (Kuge et al., 2001; Lev et al., 2005; Yang et al., 2009; Guo et al., 2011). Regulation of the AP1 protein by subcellular localization has been studied in detail in yeast and relies on the reversible binding of an export receptor, CRM1, to the YAP1 nuclear export signal (Kuge et al., 2001). We observed here that the AbAP1-eGFP fusion protein localized inside the nucleus upon exposure to ITC suggesting that AbAP1 also functions as a redox sensor that undergoes rapid activation (i.e., conformational change) due to the ITC-driven cellular redox dysregulation. As expected from this observation, the AbAP1-deficient *A. brassicicola* mutant strain was found to be highly sensitive to oxidative stress caused by H_2_O_2_, menadione and ITCs.

In mammals, ITCs have been repeatedly reported to activate parallel MAP kinase cascades, e.g., extracellular signal-regulated kinase (ERK), c-Jun N-terminal kinase (JNK) and p38 (Juge et al., 2007; Sahu et al., 2009; Geng et al., 2011; Liu et al., 2012). The MAP kinase Hog1 is the yeast homolog of p38 (Sheikh-Hamad and Gustin, 2004) and is a crucial participant in osmotic stress but has limited functions in the oxidative-stress response (Toone and Jones, 1998). By contrast, Hog1 homologs in several other fungal species have a pivotal role in the response to oxidant challenge (Moye-Rowley, 2003; Aguirre et al., 2006; Segmüller et al., 2007; Lin and Chung, 2010). Our results show that the phosphorylation status of AbHog1 changed soon after exposure to ITC and that the phosphorylated form of the protein transiently accumulated in *A. brassicicola*. In parallel we demonstrated that the MAP kinase migrated into the nucleus in fungal cells challenged with ITC. Taken together these observations strongly suggest that the MAP kinase AbHog1 is activated in response to ITC exposure. As the Δ*abhog1* mutant strains were found to be hypersensitive to oxidative stress and ITC, it could be hypothesized that the nuclear form of the MAP kinase controls the expression of a set of oxidative response genes. However, none of the ITC-induced oxidative response genes selected in our study were found to require Hog1 for induction (data not shown). In line with this observation, Enjalbert et al. (2006) showed that although inactivation of Hog1 resulted in high sensitivity toward oxidative stress in *Candida albicans*, only 46 of the 246 genes induced in response to H_2_O_2_ displayed Hog1 dependency for their induction and none of them encoded proteins with obvious antioxidant function. More recently, Heller et al. (2012) showed that the majority of gene regulated by BcSak1, the Hog1 homolog in *B. cinerea*, are not involved in the oxidative stress response. In *C. albicans*, it was also shown that mutants that lack the Hog1 MAP kinase had an enhanced basal respiratory rate, higher levels of intracellular ROS and increased sensitivity to inhibitors of the respiratory chain (Alonso-Monge et al., 2009). If such link between the Hog1 MAP kinase pathway and respiratory metabolism also exists in *A. brassicicola*, this could at least partly explain the high sensitivity of the AbHog1-deficient mutant to ITCs.

The importance of glucosinolate and glucosinolate-breakdown products in the interaction between Brassicaceae and fungal pathogens has long been a matter of debate. While some reports suggest that glucosinolate-breakdown products actively participate in plant defense (Tierens et al., 2001; Stotz et al., 2011), a correlation between glucosinolate content and resistance has not always been demonstrated (Kliebenstein et al., 2002). These apparent contradictions might reflect different lifestyles, i.e., biotrophic versus necrotrophic (Sanchez-Vallet et al., 2010), and host-ranges, i.e., broad-spectrum versus Brassicaceae-specialist (Buxdorf et al., 2013) of the pathogens as well as the variety of GLS and glucosinolate-breakdown products of the host plants. For instance, in addition to sinigrin, the precursor of Al-ITC, fifteen other GLS (aliphatic, aromatic and indolic) were identified in seeds of various Brassica species with high inter-specific or even inter-cultivar variability (Bennett et al., 2004). Our previous observations (Sellam et al., 2007a) and the data reported here demonstrated that WT strains of the Brassicaceae-specific fungus *A. brassicicola* were only slightly sensitive to various ITCs with *in vitro* IC_50_ in the millimolar range, i.e., 100- to 1000-fold higher than those measured for human bacterial pathogen (Dufour et al., 2012) and mammalian cancer cells (Liu et al., 2013), respectively. Although such high concentrations of glucosinolate-breakdown products are likely to accumulate in some parts (e.g., midvein and leaf periphery) of colonized plant tissues (Shroff et al., 2008), the ability of *A. brassicicola* to develop successful infections suggests that this fungus has evolved efficient mechanisms to overcome their toxicity. In the present study, we hypothesized that enhanced expression of oxidative response genes could represent a key mechanism for fungal protection against glucosinolate-breakdown products. *In planta* oxidative stress may originate from reactions other than the release of ITCs during infection, and the generation of ROS catalyzed by membrane-bound NADPH oxidases (Lamb and Dixon, 1997) is regarded as one of the first responses to fungal invasion (Mellersh et al., 2002). Plant inoculations were thus performed in the presence or absence of an NADPH oxidase inhibitor (diphenylene iodonium). When leaves were inoculated in the presence of the NADPH oxidase inhibitor, smaller lesions were observed irrespective of the fungal genotype. This observation is in line with previous reports showing that the oxidative burst produced by the host may facilitate colonization by necrotrophic fungi (Govrin and Levine, 2000; Williams et al., 2011; Singh et al., 2012). It is also in agreement with Pogany et al. (2009) who reported slower tissue colonization by *A. brassicicola* of the *A. thaliana* NADPH oxidase mutant *AtrbohD* compared to the WT ecotype. The reduced size of lesions on DPI-treated samples may also be explained by an inhibition of fungal NADPH oxidases that have been shown to be essential for fungal differentiation processes that are necessary for virulence (Heller and Tudzynski, 2011). Irrespective of the conditions used, Δ*abap1* and the Δ*abhog1* mutants produced significantly smaller lesions than the WT strain when inoculated on *B. oleracea* leaves. Although these observations indicate that ITCs are important in the defense of *Brassica* plants against *A. brassicicola*, it should be remembered that besides its role in the response to oxidative stress, AbHog1 has been shown to play a central role in the response to many other stresses, including phytoalexin-induced stress (Joubert et al., 2011). Similarly, although the expression of several oxidative response genes was found to be dependent of AbAP1, it has been shown that AP1 homologs may also regulate the expression of genes directly involved in pathogenicity (Guo et al., 2011). Consequently, the failure of the Δ*abhog1* and Δ*abap1* mutants to colonize *B. oleracea* leaves may not only be linked to their increased ITC sensitivity.

Recently, by comparing the behavior of the broad-spectrum pathogen *Botrytis cinerea* and the *Brassica* specialist *A. brassicicola* on different *Arabidopsis thaliana* genotypes, Buxdorf et al. (2013) suggested that *A. brassicicola* has adapted to the presence of GLS and can cope with glucosinolate-hydrolysis products more efficiently than the generalist *B. cinerea* which is more sensitive to these phytochemicals. In line with this, our results strongly suggest that the robust protection response against ICT-derived oxidative stress might be a key adaptation mechanism for successful infection of host plants by Brassicaceae-specialist necrotrophs like *A. brassicicola*. An important next step is thus to compare the cell responses to ITC in *Brassica* specialists like *A. brassicicola* and generalists like *B. cinerea*.

## Author Contributions

BC and GN contributed equally to this work. BC carried out the molecular genetic experiments, pathological tests and confocal microscopy examinations. GN and DM performed the oxygen consumption rate assays. CB and GN were involved in the phenotyping of the *Alternaria* strains and gene expression analyses. CC analyzed AbHog1 phosphorylation state. JD, SP, and ED were involved in the construction and phenotyping of strains expressing gfp and m-cherry. BI characterized ROS accumulation with the fluorescent probes. PS and TG conceived the study. All authors participated in the design of the experiments as well as analysis of the results. All authors participated in the editing and approved its final version.

## Conflict of Interest Statement

The authors declare that the research was conducted in the absence of any commercial or financial relationships that could be construed as a potential conflict of interest.

## Footnotes

1 http://rsb.info.nih.gov/ij/

2 http://genome.jgi-psf.org/Altbr1

## Supplementary Material

Figure S1 **Figure S1 | Amino-acid sequence of AbAp1**.
