# Supplementary material for: Glucosinolate-derived isothiocyanates impact mitochondrial function in fungal cells and elicit an oxidative stress response necessary for growth recovery
Source: Front Plant Sci. 2015 Jun 3;6:414. doi: 10.3389/fpls.2015.00414 (PMC4452805; doi:10.3389/fpls.2015.00414)
Supplement: Supplementary file 1 [file Table_1.DOCX]

**Table S1: List of primers used in this study**

| **Name** | | **Sequence** | | **Description** | |
| --- | --- | --- | --- | --- | --- |
| FusAP1-F1 | | ^5’^ ACCACTTCACCTCCTGTTGG ^3’^ | | Set of primers used to generate by double joint PCR the AbAP1 disruption cassette carrying the HygB resistance gene from pCB1636 | |
| FusAP1-R1 | | ^5’^*TCCTGTGTGAAATTGTTATCCGCT*AAAGAGGTCGTTGGGGTTCT ^3’^ | |  |  |
| FusAP1-F2 | | ^5’^*GTCGTGACTGGGAAAACCCTGGCG*CGGTCAAGGTGTTTTGGTCT ^3’^ | |  |  |
| FusAP1-R2 | | ^5’^ TTGACCATGATCTCCGCATA ^3’^ | |  |  |
| FusAP1-NF | | ^5’^ TTACGCCATTCCTCCTTCAC ^3’^ | |  |  |
| FusAP1-NR | | ^5’^ TTGGTCACCTTGGTGGATTT ^3’^ | |  |  |
| GfpHog1-F1  GfpAP1-F1 | | ^5’^CTTGTGTAGCACGTAGTCTGAGGT ^3’^  ^5’^ GCCTACCAACCAAGACTCCT ^3’^ | | Set of primers used to generate by double joint PCR the AbHog1- and AbAp1-Gfp fusion cassette carrying the Gfp coding sequence from pCT74 and the HygB resistance gene from pCB1636 | |
| GfpHog1-R1  GfpAP1-R1 | | ^5’^*CTCCTCGCCCTTGCTCACCAT*TCCTCCTCCGCTGCCGTTGTTCTCTTGCTCCTG ^3’^  ^5’^ *CTCCTCGCCCTTGCTCACCAT*GCCGCCGCCCCCAAGCAGTTTGTCCTTGGG ^3’^ | |  |  |
| GfpHog1-F2  GfpAP1-F2 | | ^5^’ *TCCTGTGTGAAATTGTTATCCGCT*TTACACATAATGTCTACCAATCTCG ^3’^  ^5^’ *TCCTGGTGAAATTGTTATCCGCT*CGTTCTTTCTGGGTCAACTTC ^3’^ | |  |  |
| GfpHog1-R2  GfpAP1-R2  Gfp-F1  Gfp-F2  Gfp-R | | ^5’^ CGTTGACACAACAACAACAGTCA ^3’^  ^5’^ TTGACCATGATCTCCGCATA ^3’^  ^5’^ GGCGGCGGCATGGTGAGCAAGGGCGAGGAG^3’^  ^5’^ GGAGGAGGAATGGTGAGCAAGGGCGAGGAG ^3’^  ^5’^ *GTCGTGACTGGGAAAACCCTGGCG*CTAGAGGATCCCCTTGTACAGC^3’^ | |  |  |
| Hph-F  Hph-R  AP1-F  AP1-R | | ^5’^ CGTTGCAAGACCTGCCTGAA ^3’^  ^5’^GGATGCCTCCGCTCGAAGTA^3’^  ^5’^AATGAATAGCACCGGAGTGG^3’^  ^5’^ TGAACTGAAAGACGCGTGAC^3’^ | | *Hph* and *AbAp1* specific primers for PCR validation of transformants | |
| M13F | ^5’^ CGCCAGGGTTTTCCCAGTCACGAC ^3’^ | | Set of primers used to amplify the *Hph* cassette pCB1636 | |  |
| M13R | ^5’^ AGCGGATAACAATTTCACACAGGA ^3’^ | |  |  |  |
| ExpA3H11-F1  ExpA3H11-R1  ExpA2G8-F1  ExpA2G8-R1  ExpA3G5-F1  ExpA3G5-R1  ExpA1B12-F1  ExpA1B12-R1  ExpA4D11-F1  ExpA4D11-R1  ExpA2H9-F1  ExpA2H9-R1  ExpA3D2-F1  ExpA3D2-R1  ExpA3D10-F1  ExpA3D10-R1  ExpA2F9-F1  ExpA2F9-R1  ExpA2H5-F1  ExpA2H5-R1  ExpA2C10-F1  ExpA2C10-R1  ExpA1F1-F1  ExpA1F1-R1  ExpA2C1-F1  ExpA2C1-R1  ExpA4D12-F1  ExpA4D12-R1  ExpTUB-F1  ExpTUB-R1 | | ^5’^ CCTTCCCCCCGGTATCACTA ^3’^  ^5’^ CGGCAAAGCCGTTCGA ^3’^  ^5’^ GCGGATGTGAGGGCTTTG ^3’^  ^5’^ CCAGCTCCGCAACAACCT ^3’^  ^5’^ TCGCATAACGGCGTCGAT ^3’^  ^5’^ CGTCCTAAACACACCGTTTGG ^3’^  ^5’^ TCGACATTGTAAGCGACACC ^3’^  ^5’^ TTGGAATCAGGGTGCTTCTC ^3’^  ^5’^ ACCCAAACGCCCATGCT ^3’^  ^5’^ CTGGGCCGTCGTTTTCC ^3’^  ^5’^ CCACCACATCGAGAAATCAC ^3’^  ^5’^ AACCCGGTTGATAGTGATGC ^3’^  ^5’^ GAGGAGAAATGGACGCAAAC ^3’^  ^5’^ TCCAGATTGCACAAGAGCAG ^3’^  ^5’^ GTTTCCCCTGCAATCAGTTC ^3’^  ^5’^ TCAATCTTTCCGAGGACTGG ^3’^  ^5’^ GTGACCCCATTGTCGTTTTC ^3’^  ^5’^ TGTGTTGCCAGTTGGTTGAC ^3’^  ^5’^ TGTCTCCACATCCTTTGTCG ^3’^  ^5’^ TGCACTTGTTCGTAGGATGC ^3’^  ^5’^ CCAACCACTTCAACCGTTTC ^3’^  ^5’^ TGCTGAACTTGTTGCCTACG ^3’^  ^5’^ CATGACGCTCATCAATGTCC ^3’^  ^5’^ CGTCGATTCATCATGTCCAG ^3’^  ^5’^ ATGAATGGTGCCGTTAGAGC ^3’^  ^5’^ AAGACGAGAAAGCGAAGCTG ^3’^  ^5’^ ATAACGGTGGCCCCTATTTC ^3’^  ^5’^ TCTCGCTTATGCACTTGACG ^3’^  ^5’^ TTCAACGAAGCCTCCAACAAC ^3’^  ^5’^ GTGCCGGGCTCGAGAT ^3’^ | | Sets of primers used for quantitation by real-time PCR of the relative expression of oxidative-stress responsive genes | |
|  | |  | |  | |
