# Supplementary material for: Glucosinolate-derived isothiocyanates impact mitochondrial function in fungal cells and elicit an oxidative stress response necessary for growth recovery
Source: Front Plant Sci. 2015 Jun 3;6:414. doi: 10.3389/fpls.2015.00414 (PMC4452805; doi:10.3389/fpls.2015.00414)
Supplement: Figure S1 — | Amino-acid sequence of AbAp1. [file Data_Sheet_1.DOCX]

AbAP1

1 MAGTTNDFPNGAPFYLDATQQDLLLAALASNNQNPNDLFSAGPDNSSSNKQSMINSQFQYPLDSLDPAYFTSPQQQAPAN 80

81 SFNNAGMEESPFVDYLDGDNTFDFENADGDMIGSLPGDTPGDASEKRKSPDDDGDDDDEGGGKRREGEDKQAKKPGRKPL 160

161 TSEPTT**KRKAQNRAAQRAFRERKEKHLKDLETKVQELEKASDATNHENGLLRAQVQRL**QMELREYRKRLSMNSTGVAHTP 240

241 PLAGGFSSMLNNNSNNNNFSFEFPRFGGLPGAQLLDNGPLAKNKNTSVSPGISSRHNSTGRALSPISKANGQLTNSPGPM 320

321 AGTGQRSGSLNGFFGFDSGKSNNDTPRSNTESSTGSQSRVFQFNSGSSSHSDSPSNSSTSPTNQDSS*CGTSPEPSHTSPN* 400

401 *QQVDTIKDGYVCHGNSEGEVLFCEKLNMAC*GNPRNPMPRAMSQSDDKPSPAVLAAAKPPTPAPSSASAVNGIDYFANQNG 480

481 GQFDPTLFGEYRDTQNAIVGDGDFTGGFFNDAFLNTGYGSPFHFGDTPAVQKTNPLEEIERIQDGEDEVVPGEDVDA*LLN* 560

561 *CHKIWDRLSSRPDFKDGTIDIDNLCSELRAKARCSES*GVVVDHKDVEAALKRLPKDKLLG 620

Bold : basic leucine zipper DNA-binding domain ; italics : cystein-rich domains ; underlined : nuclear export and nuclear localization sequences
